# The value-added challenge of nuts: antioxidant components, functions and applications in nut by-products

**DOI:** 10.3389/fnut.2026.1789610

**Published:** 2026-03-11

**Authors:** Yingqian Zhou, Yutong Fan, Zihan Yang, Xiaosen Han, Yuhang Li, Ting Liu, Na Sun, Tuanhui Chen, Lei Meng

**Affiliations:** 1 School of Pharmacy, Hunan University of Chinese Medicine, Changsha, China; 2 School of Biotechnology, Jiangnan University, Wuxi, China; 3 College of Biology, Hunan University, Changsha, China; 4 School of Basic Medical Sciences, North China University of Science and Technology, Tangshan, China

**Keywords:** antioxidant capacity, food applications, functional ingredients, human nutrition, nut by-products, polyphenols

## Abstract

**Objective:**

Large quantities of nut processing by products are generated worldwide and remain underutilized despite being rich sources of bioactive compounds. However, comparative integration of their antioxidant compositions and functional relevance is still limited. This review aims to summarize and compare the antioxidant compositions, functional properties, and potential food related applications of major nut by products generated during nut processing, with an emphasis on their relevance as nutritional and functional ingredients.

**Methods:**

This review was conducted as a structured narrative synthesis. Relevant literature was retrieved from major academic databases, including Web of Science, Scopus, PubMed, and China National Knowledge Infrastructure (CNKI), covering publications from 2000 to 2025. Earlier foundational studies were also included when necessary to provide historical context. Search terms combined the names of major nut by-products with keywords related to polyphenols, flavonoids, polysaccharides, and antioxidant activity. Studies were selected based on relevance to compositional characterization, antioxidant activity evaluation, mechanistic investigation, and application-related research. Both international and regional peer-reviewed publications were considered to provide a comprehensive overview of current research progress.

**Results:**

Nut by products are rich in bioactive compounds such as polyphenols, flavonoids, and polysaccharides, which exhibit strong antioxidant capacity through scavenging reactive oxygen species and modulating oxidative processes. Comparative analysis indicates notable differences in antioxidant efficacy and functional performance among different nut residues. Beyond antioxidant activity, these by products show promising potential in food preservation, freshness maintenance, and natural coloring, as well as nutrition related functionalities including antimicrobial and anti inflammatory effects.

**Discussion:**

The findings highlight nut by products as valuable sources of antioxidant compounds for food and nutrition related applications. However, challenges including compositional variability, extraction efficiency, safety evaluation, and standardization remain key barriers to their large scale utilization. Addressing these issues may facilitate the development of value added functional ingredients and support the sustainable utilization of nut processing by products within food systems, thereby promoting circular economy strategies and enhancing the overall value chain of nut industries.

## 1 Introduction

Nuts are classified as closed fruits, characterized by their hard shells and seeds. These nutritious fruits are widely recognized for their favorable nutritional profiles, being rich in protein, oils, minerals, vitamins, and other essential nutrients that contribute to overall health maintenance. Research indicates that consuming nuts more than twice a week can lower the risk of fatal heart disease and may also aid in cancer prevention. The five types of nuts discussed—walnuts, chestnuts, cashews, peanuts, and almonds—are commonly cultivated in China, where they are known for their rich nutritional content and unique flavors. These nuts have substantial value for their comprehensive development and utilization. However, much of the nut production often results in waste; the rinds, shells, and other by-products are frequently discarded as household garbage. This not only leads to a significant loss of potential resources but also contributes to environmental issues. According to national agricultural statistics, China discards approximately 4.5 million tons of green walnut peel and 5.1 million tons of peanut shells each year (1, 2). Given that China is one of the world’s largest nut producers, these figures illustrate the considerable scale of nut processing residues generated globally. Addressing this waste could enhance resource efficiency and reduce environmental impact.

Reactive oxygen species (ROS) are small molecules derived from oxygen and are by-products of normal cellular metabolism. Under normal physiological conditions, the generation and elimination of reactive oxygen radicals maintain a dynamic equilibrium essential for vital processes such as cell signaling transduction and proliferation. When excessive ROS production exceeds the effective capacity of cellular antioxidants, oxidative stress occurs, disrupting redox homeostasis and impairing cellular components such as proteins, DNA, RNA, and lipids. In this context, natural antioxidants derived from food resources, including nut by-products rich in phenolic compounds, have attracted increasing attention for their potential to mitigate oxidative processes.

Antioxidants available in the market are primarily categorized into two groups: natural and synthetic. Some studies have raised safety concerns regarding the potential adverse effects of certain synthetic antioxidants under specific conditions or at high intake levels. As a result, natural antioxidants are increasingly being explored as alternative options. Nut by-products, in particular, have been identified as rich sources of various flavonoids, polyphenols, and polysaccharides, all of which exhibit significant antioxidant properties and high practical value. In the food industry, these natural antioxidants can prevent spoilage, inhibit bacterial growth, and extend the shelf life of products. In the field of medicine, they play a crucial role in scavenging excess free radicals, thereby maintaining a balance within the body to mitigate the harmful effects of oxidative stress. This also helps to enhance vitality, boost immune function, reduce inflammation, and inhibit tumor growth. Moreover, in industrial applications, these natural antioxidants can be utilized as raw materials in dye additives and other products, addressing the limitations of synthetic dyes while enhancing effectiveness.

This paper focuses on several representative by-products derived from nuts, including green walnut peel, peanut shells, chestnut shells, almond peel, and cashew nut shells. While these materials have been individually investigated in previous studies, a comprehensive and comparative synthesis of their antioxidant compositions and application potential remains limited. By examining these by-products within a unified analytical framework, this review aims to provide a clearer understanding of their relative characteristics and functional value.

**Figure 1:**
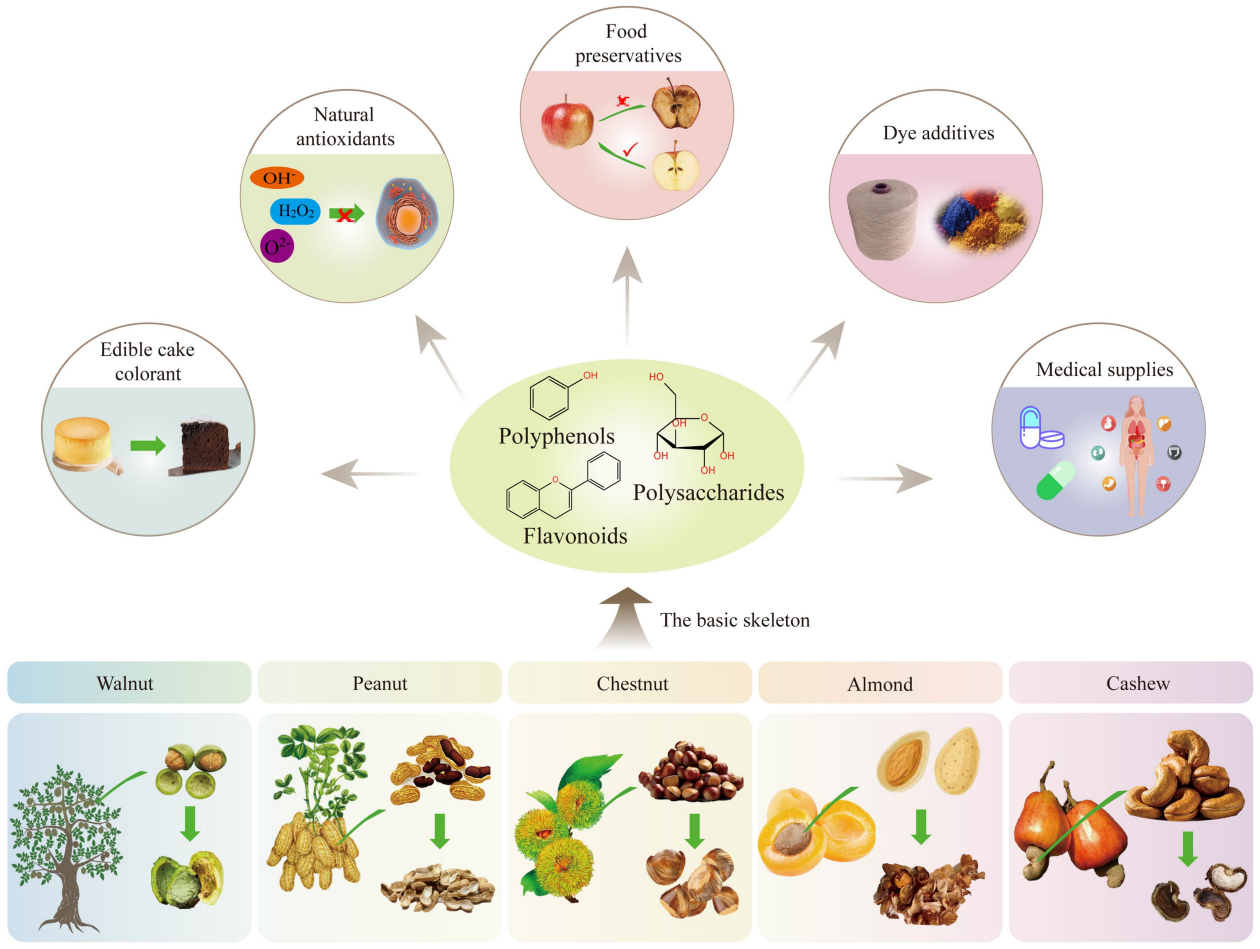
Structural diagram of various parts of the nut.

**Figure 2:**
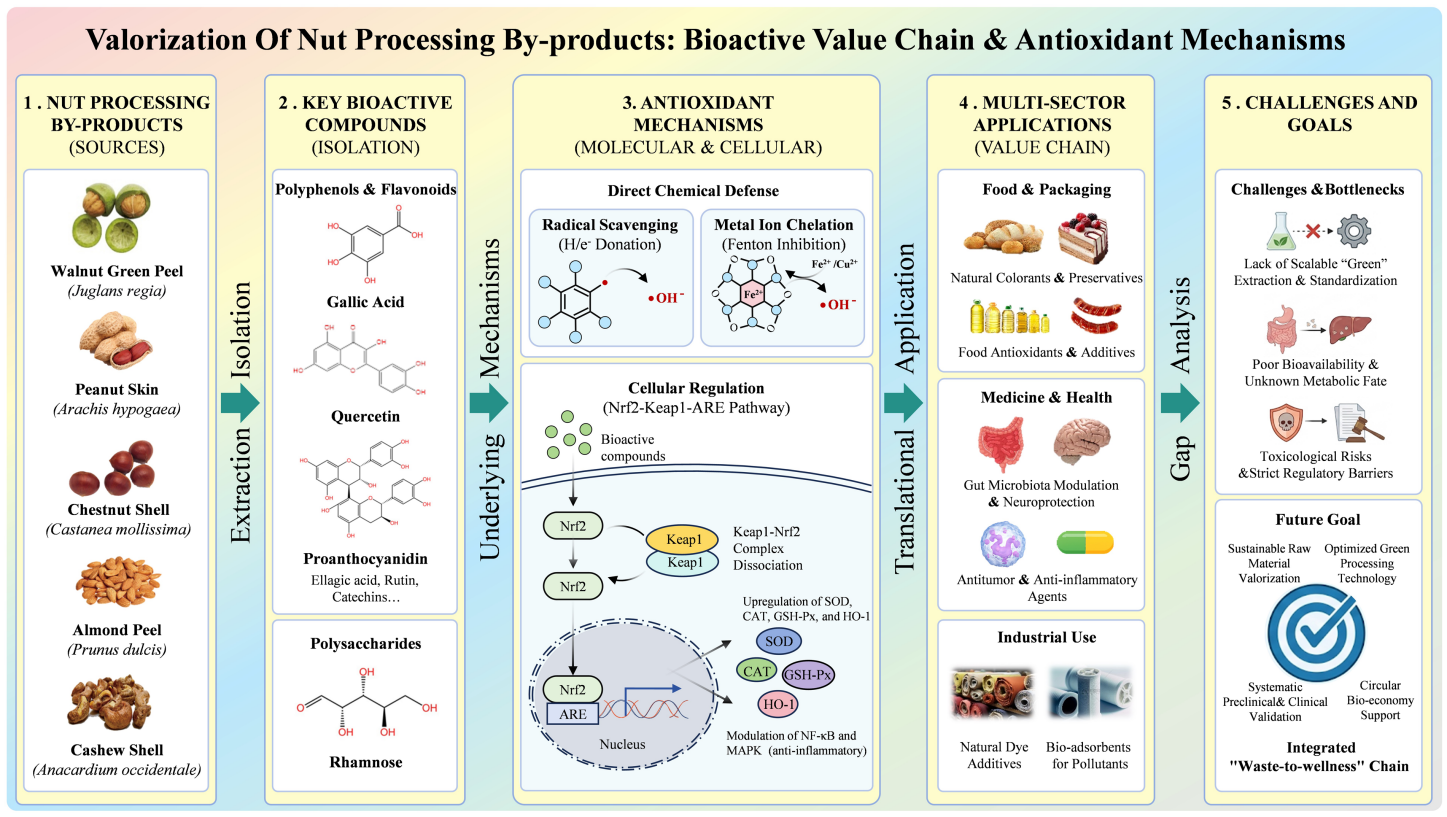
Valorization of nut processing by-products: bioactive value chain & antioxidant mechanisms.

Specifically, this paper systematically summarizes the antioxidant constituents of these nut by-products and comparatively evaluates their reported efficacy and practical applications in food, nutrition, and industrial contexts. By integrating compositional data with functional performance and utilization strategies, this review offers an updated and consolidated perspective to support value-added utilization and sustainable management of nut processing residues. The conceptual framework of this review is illustrated in Figures 1, 2.

## 2 Core components of nut by-products that exert antioxidant properties

Polyphenols, flavonoids, and polysaccharides are the main antioxidant components found in nut by-products. Table 1 provides an overview of their distribution across different types of residues. While these compound classes are present in most nut matrices, their relative amounts and compositional profiles vary considerably depending on the plant source and how the material was processed. Table 2 presents representative molecular structures and reported functional properties of selected bioactive compounds, emphasizing the structural features that contribute to antioxidant activity.

**Table 1:** Ingredients in various nuts.

Nut types	Major phenolic acids	Major flavonoids	Tannins/Others	Reference
Green Walnut Peel	Gallic acid; Chlorogenic acid; Ferulic acid; Caffeic acid; 4-hydroxycinnamic acid; Vanillic acid; p-Hydroxybenzoic acid; Salicylic acid	Rutin; Kaempferol; Quercetin; Hyperin; (2 s)-5,7,4′-trihydroxydihydroflavone; (2 s)-5-hydroxy-6,7-dimethoxydihydroflavone; 5-hydroxy-3,3,3,4′-tetramethoxyflavone; 5-hydroxy-3,3,3,4′-tetramethoxyflavone	Ellagic acid; Tannic acid; Citric acid; Carotenoid glycosides; Betaine;	(11–18, 46)
Chestnut shells	Gallic acid; Protocatechuic acid; Chlorogenic acid; Ferulic acid; ρ-Coumaric acid; Vanillinic acid; p-Hydroxybenzoic acid	Rutin; Kaempferol; Catechins; Quercetin; Apigenin; Naringin;	Proanthocyanidins; o-Dipheno; Ellagic acid; Lignans; Butyric acid	(4, 5)
Cashew nutshell liquid	Anacardic acid; Cashew acid;	—	Cardanol; Cardol; Phytosterols; Tricosanoids	(27, 28)
Peanut shells	—	Luteolin; 5,7-dihydroxychromanon;	—	(35, 36)
Almond skins	—	Flavonoids (general)	—	(43)

**Table 2:** Specific structure and function of some components.

Categorization	Chemical composition	Structural formula	Efficacy	Reference
Polyphenol	Gallic acid	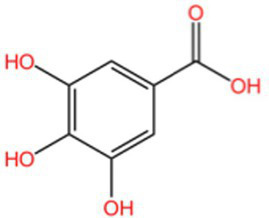	Antioxidant Anti-inflammatory Anti-tumour Antiviral Antibacterial	(92) (93)
	Ethyl gallate	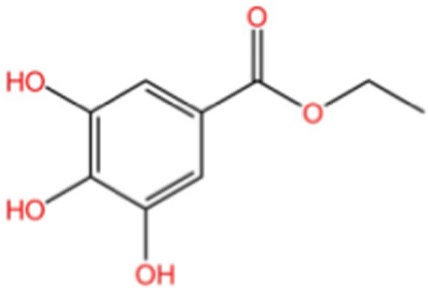	Antioxidant Anti-inflammatory	(94)
	4-Hydroxybenzoic acid	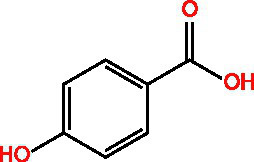	Preservative Antimicrobial Antioxidant Antibacterial activity	(11)
	Vanillic acid	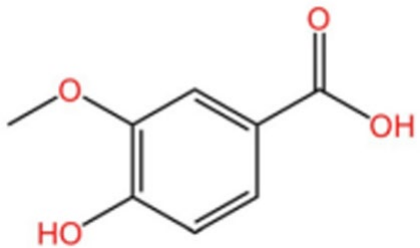	Antimicrobial Anti-inflammatory Antioxidant Flavouring ag	(95) (12)
	Vanillinic acid	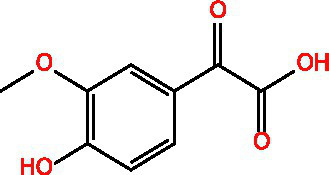	Antioxidant	(4, 5)
	Anisic acid	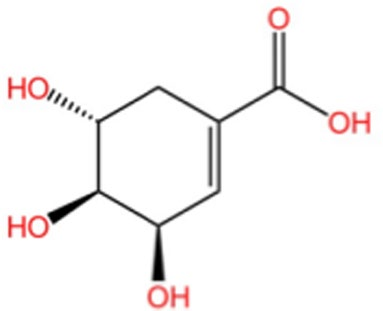	Antibacterial anti-inflammatory Antioxidant	(96)
	Protocatechuic acid	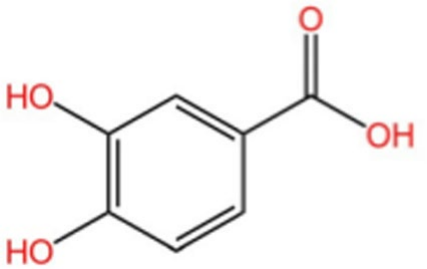	Reduce myocardial oxygen consumption Improving myocardial oxygen tolerance Antioxidant	(97)
	Salicylate	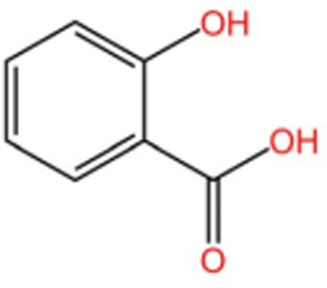	Antioxidant	(98)
	Gentianic acid	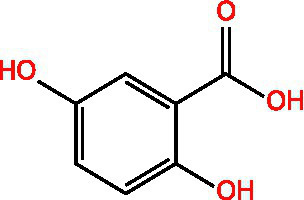	Antioxidant	(4, 5)
	Chlorogenic acid	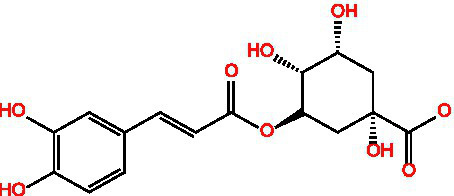	Antibacterial Antioxidant Anti-inflammatory Antivirus	(99) (11)
	Ferulic acid	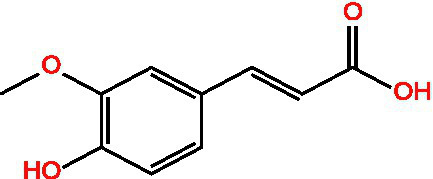	Antioxidant Antithrombotic Anti inflammatory	(100) (11)
	Eugenic acid	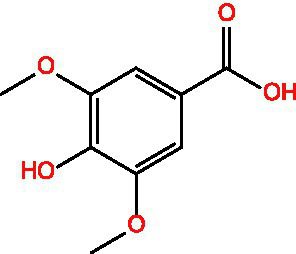	Antioxidant Neuroprotection Antibacterial activity	(4, 5) (11)
	Scopolamine lactone	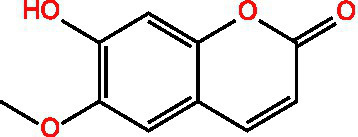	Antioxidant Anti inflammatory Anti cancer	(101)
	Caffeic acid	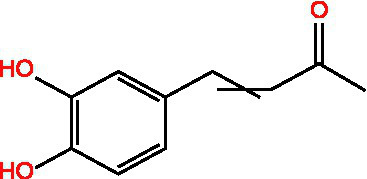	Antioxidant	(102)
	Mustard acid	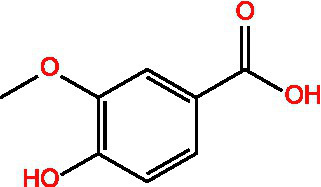	Antioxidant Antibacterial activity Anti cancer Anti anxiety	(103)
	3-Caffeoylquinine	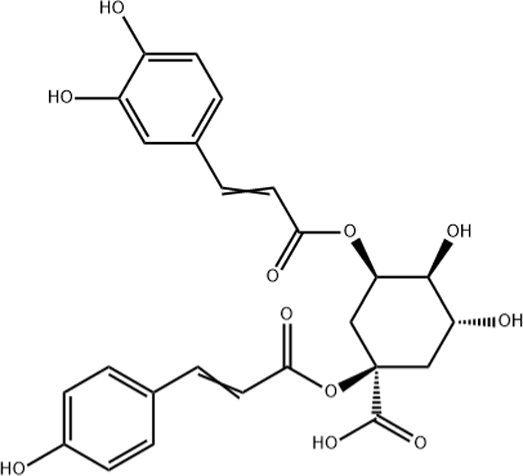	Antioxidant	(13)
	3-Acetylphenol	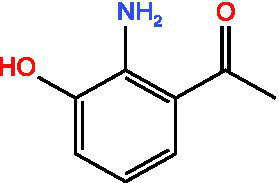	Antioxidant	(13)
	Cashew acid	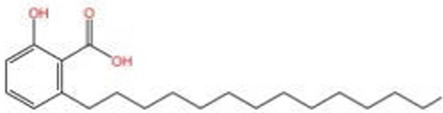	Anti cancer Antibacterial activity Antioxidant	(27, 28)
	Cashew nut phenol	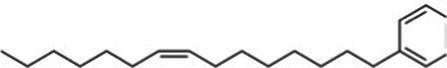	Antioxidant Anti-inflammatory	(104)
Flavonoids	Rutin	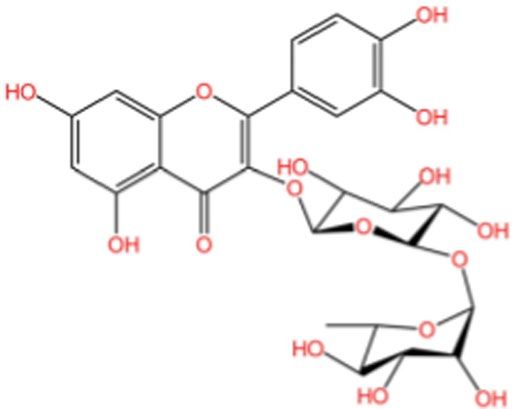	Anti-free radicals Antilipid peroxidation	(105) (15)
	Kaempferol	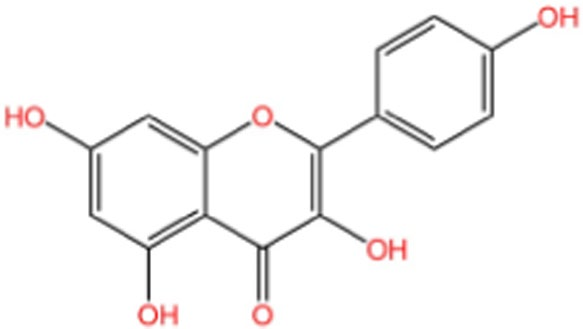	Antioxidant Anti inflammatory	(106) (15)
	Isorhamnetin	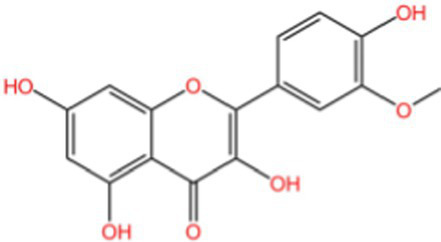	Antioxidant	(107)
	Rhinocerosin	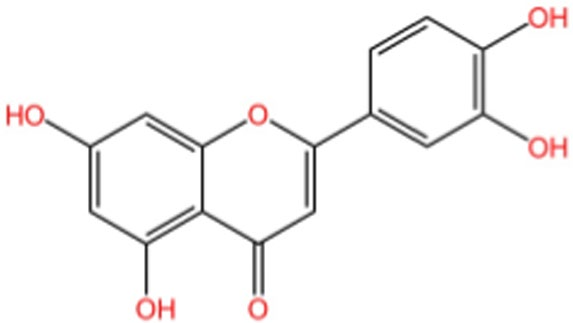	Antioxidant Anti inflammatory Anti diabetes Anti cancer	(108) (35, 36)
	Apigenin	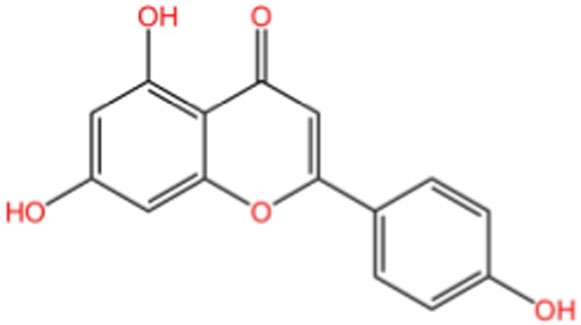	Antioxidant Anti metastasis	(109)
	Naringin	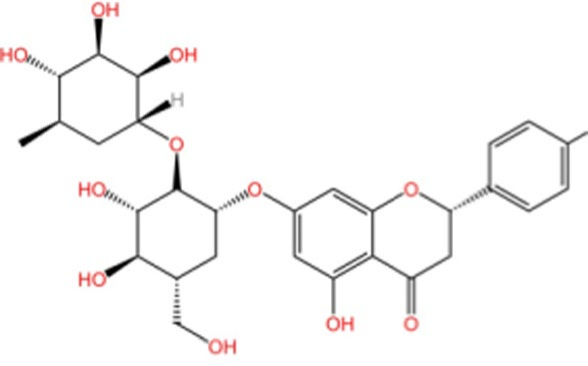	Antioxidant Anti inflammatory Antitumor Improve sugar and lipid metabolism	(110)
	Quercetin	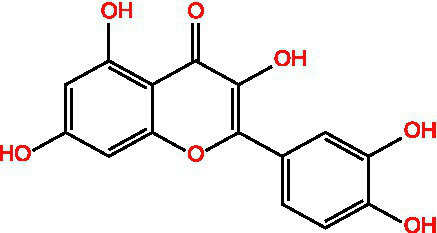	Antioxidant Neuroprotection	(111) (15)
	Catechuic acid	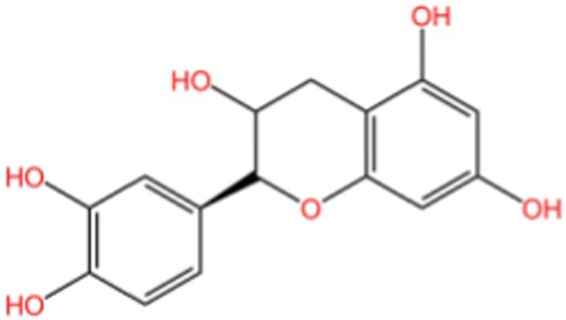	Anti cancer Clearing free radicalsAntioxidant Antibacterial activity	(112)
	Epicatechin	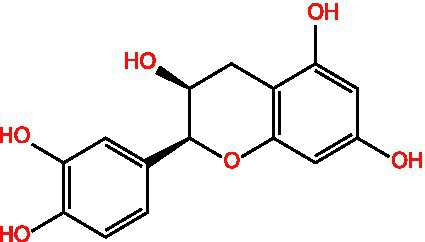	Antioxidant Anti inflammatory Antibacterial activity	(113)
	Epigallocatechin	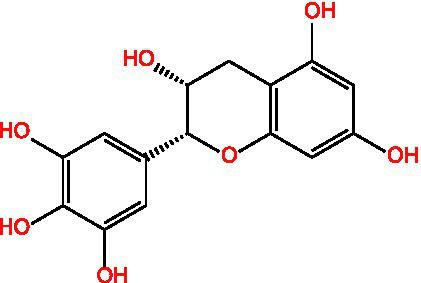	Antioxidant	(4, 5)
	Flavonol	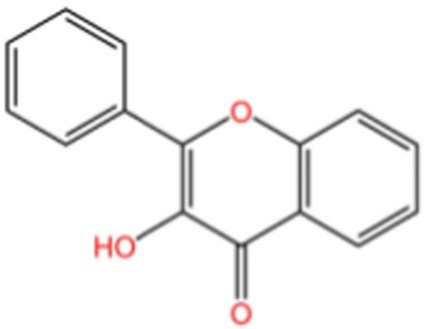	Clearing free radicals Antioxidant	(114)
	Carotene	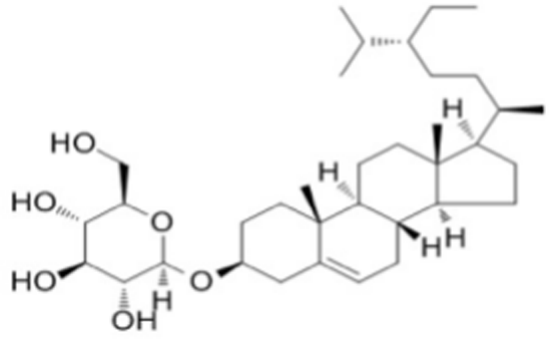	Antioxidant Antitumor	(115)
	Hyperin	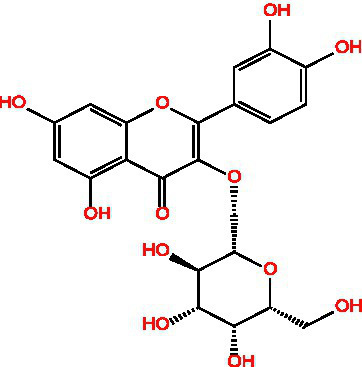	Antioxidant	(15)	(2 s)-5-Hydroxy-6,7-dimethoxydihydroflavone	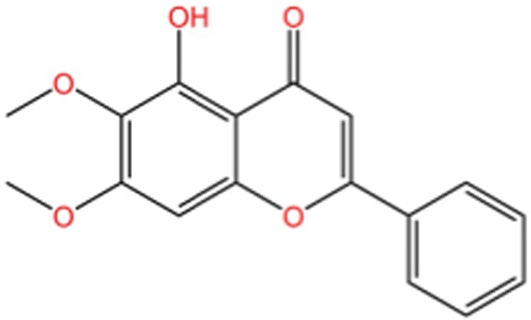	Antioxidant	(15)	4-[3’-Hydroxy-1′-propenyl]-2-methoxyphenol	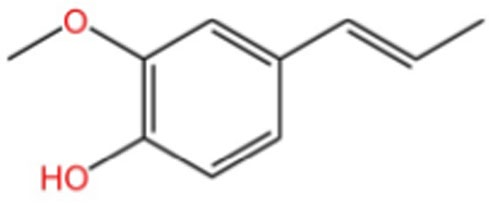	Antioxidant	(15)	5,7-Dihydroxychromanone	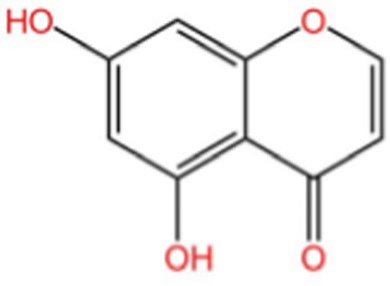	Antioxidant	(35, 36)	Holocaust phenol	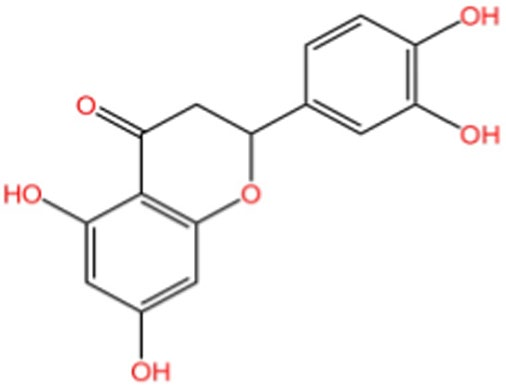	Antioxidant Anti inflammatory Antitumor Neuroprotection	(49)	Tannic acid	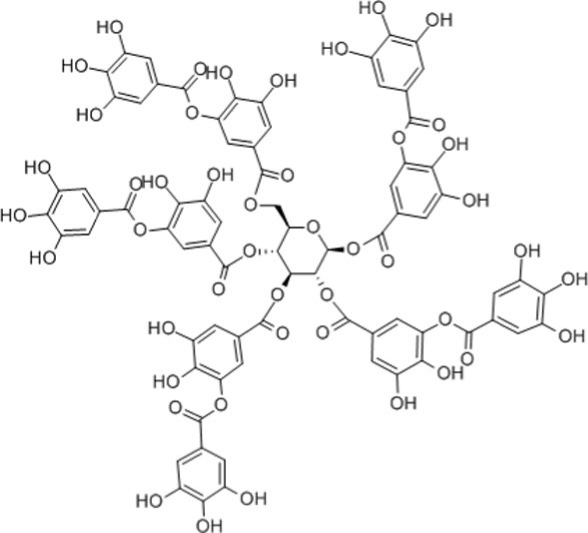	Antioxidant Antibacterial activity	(16)
	Tannin	Ellagic acid	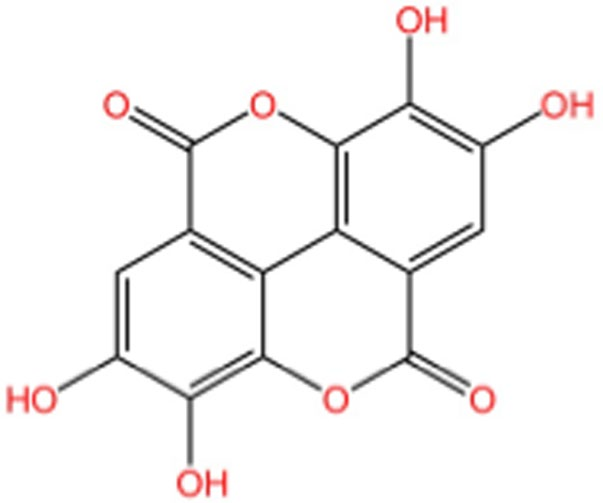	Antioxidant	(116) (16)
		Proanthocyanidin	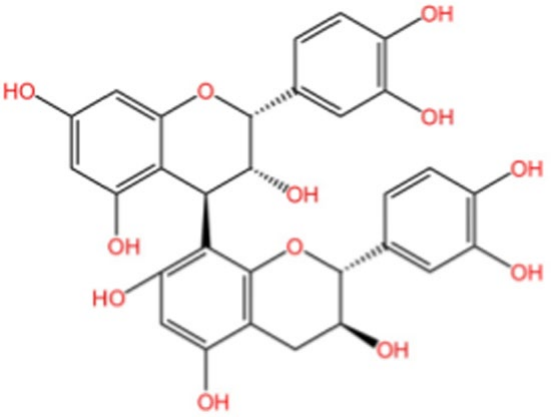	Antioxidant Anti inflammatory Anti cancer Lowering blood sugar	(117)
	Polysaccharide	Rhamnose	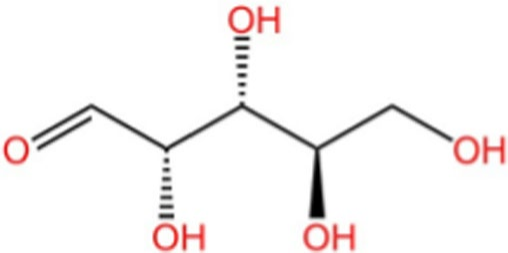	Antioxidant Cellular protection	(118)
		Arabinose	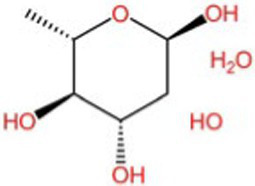	Antioxidant	(119)

Rather than relying on a single mechanism, these compounds act through multiple antioxidant pathways. These include scavenging free radicals, chelating metal ions, inhibiting lipid peroxidation, and modulating endogenous antioxidant enzymes. Comparative analysis indicates that phenolic-rich residues, such as chestnut shells and walnut green peels, tend to exhibit stronger radical-scavenging activity *in vitro*. In contrast, polysaccharide fractions may play a more significant role in biological antioxidant responses, often through indirect regulatory mechanisms. However, variability in extraction and analytical methods limits direct cross-study comparability, highlighting the need for standardized evaluation frameworks. In the following sections, we integrate compositional data with functional outcomes to highlight both common trends and material-specific characteristics across nut by-products.

### 2.1 Polyphenols

Plant polyphenols are a class of secondary metabolites with polyphenolic structure widely present in plants, mainly in the skin, roots, leaves and fruits. They exhibit multiple physiological functions, such as antioxidant, antibacterial, antitumor, antiviral and antiallergic activities, and are known as the “health guardians” of nature and humans (3). Studies have shown that nut by-products like chestnut shells and walnut green husks contain diverse and high-content polyphenols, with strong antioxidant effects.

#### 2.1.1 Chestnut shells

Polyphenolic compounds in chestnut shells are mainly classified into phenolic acids, flavonoids, and tannins, as summarized in Table 1. Phenolic acids such as gallic acid and chlorogenic acid are frequently reported as dominant constituents. Representative flavonoids include quercetin, rutin, and catechin, while tannins are primarily composed of ellagic acid and proanthocyanidins. In addition, o-diphenol has been identified as a relatively abundant phenolic component in chestnut shells (4, 5).

There is a good correlation between phenolic compounds in plants and antioxidant ability. Yao et al. (6) performed fractionation analysis and observed marked differences among solvent-separated components: Fr.3 exhibited the strongest activity (IC₅₀ = 66.5 ± 1.0 mg·L^−1^), followed by Fr.2 (75.5 ± 2.1 mg·L^−1^) and Fr.1 (292.2 ± 3.9 mg·L^−1^), indicating that moderately polar fractions are enriched in active phenolics. Consistently, Li et al. (7) found that extracts obtained using moderately polar solvents exhibited stronger reducing power and superoxide anion scavenging activity, while Wang et al. (8) showed that ultrasonic-assisted aqueous extracts possessed robust multi-radical scavenging capacity. Extending these findings to different anatomical parts, Silva et al. (9) conducted a comparative evaluation across chestnut by-products and reported EC₅₀ values of 0.06 ± 0.01 mg/mL for inner shell and 0.12 ± 0.02 mg/mL for outer shell, demonstrating substantial intra-species variability.

#### 2.1.2 Walnut green peel

Walnut green husks contain diverse phenolic acids, flavonoids, and hydrolysable tannins, as summarized in Table 1. The total phenolic content has been reported to be approximately 21.71 ± 0.98 mg/g (10). Major phenolic acids include ferulic acid and caffeic acid derivatives (11–14), while flavonoids are predominantly represented by flavonols and their glycosides, such as rutin and quercetin (15). Hydrolysable tannins, including ellagic acid and tannic acid, constitute a substantial portion of the phenolic profile (16). Notably, reported tannin levels vary considerably (4–19%) depending on analytical methodology and geographical origin (17, 18).

Hu et al. (19) found that walnut green husks extract could inhibit lipid peroxidation by scavenging ·OH. Fernández-Agulló et al. (20) extracted walnut green husks using solvents of different polarities and evaluated their antioxidant activities. The results showed that walnut green husks ethyl acetate and chloroform extracts had the strongest antioxidant capacity, with total phenol content of 193.52 mg/g and 174.64 mg/g, respectively, DPPH scavenging capacity of 14.05 μg/mL and 26.11 μg/mL, respectively and reducing capacities of 34.70 μg/mL and 44.60 μg/mL, respectively, including broad antioxidant activities. Romano et al. (21) used supercritical CO₂ extraction of walnut green husk polyphenols and demonstrated that the extract enhanced superoxide dismutase (SOD) activity, indicating strong *in vivo* antioxidant efficacy.

#### 2.1.3 Peanut shells

Tran et al. (22) reported DPPH and hydroxyl radical scavenging rates of 88.5 and 93.6%, respectively, following cellulase-assisted ethanol extraction of peanut shell polyphenols. Broader quantitative analyses indicate that peanut shell extracts typically exhibit DPPH scavenging rates of 30–90% within 1–5 mg/mL, with EC₅₀ values commonly ranging from 0.8–1.5 mg/mL for ethanol extracts and 1.8–3.0 mg/mL for aqueous extracts (23), suggesting superior efficiency of moderately polar systems. Under optimized conditions, scavenging capacity may exceed 85%, approaching the activity of vitamin C (≥95% at 50 μg/mL). Additionally, structural modification of soluble dietary fiber (SDF) via PEF-TPP treatment reduced DPPH IC₅₀ to 4.42 mg/mL compared with 5.54 mg/mL in alcohol-precipitated SDF (24), indicating enhanced antioxidant efficiency.

#### 2.1.4 Almond skins

Szymanowska et al. (25) reported that almond skin exhibits DPPH radical scavenging activity dependent on extraction conditions. In buffered extracts, activity increased from approximately 5–8 to 15–18 μg Trolox/mL with increasing addition levels, whereas ethanol extracts showed higher values (22–25 μg Trolox/mL), indicating greater efficiency of organic solvents. The same study also found that simulated gastrointestinal digestion markedly enhanced antioxidant capacity (≈140–148 μg Trolox/mL), suggesting improved release of phenolic compounds. Picerno et al. (26) investigated the antioxidant activity of almond skin aqueous extracts, reporting SC₅₀ values of approximately 211.6 μg/mL, which slightly increased after storage. Notably, microencapsulation reduced the SC₅₀ to around 202 μg/mL and improved stability. Although weaker than pure catechin, almond skin demonstrates consistent and formulation-responsive antioxidant activity.

#### 2.1.5 Cashew nutshell liquid

Cashew nut shell liquid (CNSL), is a dark brown oily liquid extracted from the outer shell of the tropical plant *anacardium occidentale*, which is highly corrosive and erodes the skin, and the main components are cashew acid (which is very easy to deacidify to cardanol), cardanol, cardol, 2-methylcardiol, phytosterols, tricosanoids, and anacardic acid (27, 28), of which cardanol, constitutes 60 to 90% of the total content (28).

Crude CNSL is highly corrosive due to the presence of anacardic acid and related phenolic lipids, which can cause skin irritation and chemical burns. This inherent reactivity raises safety concerns regarding its direct application, particularly in food or biomedical contexts. However, decarboxylated derivatives such as cardanol, as well as purified fractions, exhibit reduced corrosiveness and have been investigated for antioxidant and polymer-related applications (29, 30). Several studies have reported that CNSL and its phenolic constituents possess DPPH radical scavenging activity, although the activity is generally weaker than classical antioxidants such as ascorbic acid and rutin (27). Therefore, while CNSL demonstrates antioxidant potential, its practical applicability depends on purification, structural modification, and careful evaluation of toxicity and regulatory compliance.

#### 2.1.6 Comparative evaluation

A comparative analysis reveals a distinct antioxidant hierarchy, with walnut green peels and chestnut shells demonstrating superior potency over peanut and almond residues. While a consensus exists regarding the higher efficiency of moderately polar solvents across the species examined, it is imperative to note that the quantitative values reported in various studies, such as IC₅₀ and scavenging percentages, serve only as relative benchmarks rather than absolute standards of superiority. The absence of a standardized evaluation framework, including consistent reference antioxidants (e.g., Trolox), uniform reaction times, and standardized solvent-to-solid ratios, makes direct cross-study comparisons methodologically tenuous. Furthermore, literature reveals significant contradictions regarding processing; for instance, while storage typically degrades activity, simulated digestion can paradoxically enhance almond skin potency through phenolic release. This safety-efficacy paradox, exemplified by the corrosiveness of cashew nut shell liquid, further highlights that practical applicability depends more on purification and physiological stability than on raw radical-scavenging capacity measured in chemical assays.

### 2.2 Flavonoids

Flavonoids generally refer to a series of compounds in which two benzene rings with phenolic hydroxyl groups are connected by a central three-carbon chain. They are widely present in the plant kingdom, serving as an important class of natural substances in plants, and exhibit functions such as antioxidant, anti-inflammatory, antibacterial, antiviral, and antitumor activities.

#### 2.2.1 Chestnut shells

Chestnut shells contain abundant natural brown pigments. Most studies have shown that its main active components are flavonoids (31), which have good antioxidant activity and can eliminate free radicals, block and scavenge oxidative damage, antibacterial,anti-inflammatory, and delay aging (32).

Li et al. (33) studied the antioxidant activities of chest nut shell pigment in lard and found that its antioxidant properties were superior to those of BHT and Vitamin E (VE). Li et al. (34) studied the antioxidant activities of chest nut shell browning pigments extracted by different solutions. The results showed that both alkali-extracted and alcohol-extracted chestnut shell brown pigments exhibited certain antioxidant activities. Gao et al. (31) experimentally investigated the reducing and antioxidant capacities of chestnut husk browning pigment. The results showed that the reducing ability of chestnut husk browning pigment was comparable to that of VC at concentrations higher than 1.8 mg/mL, and that the antioxidant capacity was strong and increased with the increase in concentration.

#### 2.2.2 Peanut shells

Peanut shells contain flavonoids such as luteolin, 5,7-dihydroxychromanone, and eriodictyol, of which luteolin is the most abundant (35, 36). Its main mechanism is to terminate the free radical chain reaction by reacting phenolic hydroxyl groups with free radicals to generate relatively stable half-roll radicals (37).

Yu et al. (38) studied the antioxidant effect of ethanol extracts from peanut shells at different concentrations on lard and found that the antioxidant effect increased with concentration. Yan et al. (39) studied the antioxidant activity of luteolin and found that when luteolin were added at a dose of 0.02%, the antioxidant effect was comparable to that of BHT at the same dose and better than that of tea polyphenols at the same dose. The reducing power and the ability to scavenge hydroxyl radicals were better than that of tea polyphenol and BHT. Sun et al. (40) found that flavonoids in peanut shells had significant antioxidant effects on lard, and the antioxidant effect was similar to that of 0.2% BHT when the additive amount was 0.2%. Zhang et al. (41) investigated the *in vitro* antioxidant activity of peanut shell extracts through a variety of systems, and found that peanut shell extracts had strong scavenging ability for three free radicals, DPPḤ, ·OH, and O₂^−^·, and within a certain range, increased with the increase in concentration. Zhou et al. (42) experimentally found that the flavonoids in peanut shells had a strong scavenging capacity for DPPḤand ABTS+· radicals, and the scavenging rate was positively correlated with the flavonoid content. Both the total antioxidant capacity and the reducing power were higher than those of synthetic antioxidants.

#### 2.2.3 Almond skins

Almond skins, the reddish-brown or dark yellow seed coats of almonds, contain various active substances such as cellulose and flavonoids. Wang et al. (43) found that flavonoids in almond skins exhibited strong scavenging abilities against DPPḤ and ·OH free radicals, as well as significant inhibition of lipid peroxidation, but showed weak scavenging capacity for O₂^−^·.

#### 2.2.4 Walnut green peel

Total flavonoids from walnut green peels have a variety of biological activities. Lu et al. (44) used Box–Behnken design response surface test to optimize the extraction process of total flavonoids from walnut green peels and conducted *in vitro* antioxidant activity studies on them. The result showed that total flavonoid extract from walnut green peels with a mass concentration of 1.0 g/L showed a 94.98% scavenging rate of DPPḤ and ·OH free radicals, respectively, 89.89%, which was slightly lower than the antioxidant capacity of VC at the same mass concentration, but still showed better antioxidant activity.

Although flavonoids consistently exhibit concentration-dependent antioxidant activity, their efficacy is strongly influenced by hydroxylation patterns and glycosylation status. For instance, luteolin-rich peanut shells display potent chain-breaking antioxidant behavior, whereas flavonoid glycosides in walnut peels may show comparatively lower direct radical-scavenging efficiency, yet offer improved solubility and bioavailability. This structure–function relationship highlights that antioxidant activity is determined not only by total flavonoid content, but also by molecular configuration.

### 2.3 Polysaccharides

Polysaccharides are made of more than 10 monosaccharides linked by glycosidic bonds, which are important components of living organisms and one of the four basic substances constituting life. Most polysaccharides have strong biological activities, such as antioxidant, antitumor, antiviral, anti-radiation, hypoglycemic, lipid-lowering and hepatoprotection, etc. Among them, walnut green husks polysaccharides are also able to improve the immune function of erythrocytes (45). Ji et al. (46) measured the crude polysaccharide content in walnut green peels as 38.07% and refined polysaccharide content as 76.08% by aqueous ethanol precipitation and high performance capillary electrophoresis, respectively. The monosaccharide composition mainly includes galactose (42.998%), glucose (23.30%), arabinose (16.03%), rhamnose (10.123%), and fructose (7.549%).

Some scholars have experimentally demonstrated that walnut green husk polysaccharide has some reducing ability to scavenge ·OH, O₂-·, and DPPH free radicals, which is enhanced with increasing concentration, but its effect is lower than VC (47). Lv et al. (48) found that 1.0 g/L of walnut green husk polysaccharide (extraction yield: 9.02%) scavenged 58 and 57% of ·OH and O₂-·, respectively. The ability of walnut green husk polysaccharide to scavenge ·OH and O₂-·was determined by salicylic acid method and pyrogallol autoxidation method, and it was found that the scavenging ability of walnut green husk polysaccharide was weaker than VC; walnut green husk polysaccharide has total antioxidant ability, and its ability of scavenging O₂-· radicals, DPPḤ radicals, and ABTS· radicals was weaker than VC.

Although walnut green husk polysaccharides exhibit comparatively weaker direct radical-scavenging activity than phenolic compounds in chemical assays, their biological antioxidant effects may involve alternative mechanisms, including modulation of endogenous antioxidant enzymes and immune regulation. In addition, polysaccharides generally present advantages such as lower cytotoxicity and better biocompatibility. Therefore, their functional value should not be assessed solely based on *in vitro* radical-scavenging capacity. Walnut green husk polysaccharides may hold potential as functional food ingredients or nutraceutical components, particularly in applications where safety and immunomodulatory effects are prioritized.

### 2.4 Structural–functional hierarchy and antioxidant contribution patterns

Across the examined nut by-products, antioxidant efficacy follows a distinct structural–functional hierarchy that dictates their industrial application. Phenolic compounds, particularly low-molecular-weight phenolic acids and flavonoids, primarily contribute to rapid free radical scavenging and metal-chelating activity in chemical systems. In contrast, polymeric tannins and complex polysaccharides exhibit more moderate direct scavenging capacity but offer broader biological relevance through the modulation of endogenous antioxidant enzymes and cellular redox signaling.

However, transitioning these residues from laboratory-scale extracts to validated functional ingredients requires addressing three critical pillars. First, industrial scalability remains limited by the lack of standardized, “green” extraction frameworks that ensure bioactive consistency across different batches. Second, current evaluations must move beyond chemical assays to account for the bioavailability and metabolic fate of these compounds within the gastrointestinal tract, as physiological efficacy is often governed by metabolic transformation rather than raw antioxidant power. Finally, comprehensive safety assessments are a prerequisite for regulatory approval, particularly for materials with inherent toxicity risks, such as cashew nut shell liquid.

In conclusion, recognizing this structural-functional stratification—combined with rigorous validation of safety and *in vivo* stability—is essential for the targeted valorization of nut processing by-products within a sustainable and circular food economy.

## 3 Antioxidant mechanisms of the core components

Polyphenols, flavonoids, and related bioactive compounds derived from nut by-products exhibit overlapping antioxidant mechanisms. These mechanisms can be broadly categorized into: (1) direct radical scavenging through hydrogen atom or electron transfer; (2) transition metal ion chelation; (3) inhibition of pro-oxidant enzymes; (4) activation of endogenous redox-regulatory pathways such as Keap1-Nrf2-ARE. The relative contribution of each mechanism is largely determined by molecular structure, degree of polymerization, and bioavailability. Rather than reiterating these shared mechanisms in each subsection, Table 3 summarizes the common and compound-specific antioxidant actions.

**Table 3:** Comparative synthesis of bioactive components from nut by-products Structural basis, mechanistic pathways, and functional applications.

Chemical class	Representative compounds	Major nut by-product sources	Primary mode of antioxidant action	Secondary mechanisms	Application relevance
Phenolic acids	Gallic acid	Walnut green husk, Chestnut shell	Direct radical scavenging via hydrogen donation	Mild metal chelation; partial Nrf2 activation	Natural preservatives; functional food ingredients
	Chlorogenic acid	Peanut skin, Almond skin	Radical scavenging and metal chelation	Redox signaling modulation; mitochondrial protection	Nutraceuticals; metabolic health
	Ferulic acid	Peanut shell, Almond by-products	Chain-breaking antioxidant via phenoxy radical stabilization	Enzyme inhibition; Nrf2-mediated enzyme upregulation	Food stabilization; dermatological applications
Condensed tannins	Oligomeric proanthocyanidins	Walnut green husk	Potent radical scavenging and Fe^2^⁺/Cu^2^⁺ chelation	NADPH oxidase inhibition; Nrf2 activation; anti-inflammatory signaling	Cardioprotective; anti-inflammatory nutraceuticals
Flavonols	Quercetin	Peanut skin, Almond skin	Multi-pathway redox modulation	Metal chelation; enzyme inhibition; PI3K/Akt and MAPK regulation	Functional foods; neuroprotective potential
	Rutin	Chestnut shell, Peanut skin	Lipid peroxidation inhibition	Enhancement of SOD and CAT activity	Food preservation; vascular protection
	Hyperin	Almond skin	Mitochondrial stabilization and ROS reduction	Nitric oxide synthase modulation	Cardioprotective applications
Flavones	Apigenin	Peanut residues	Radical scavenging and signaling modulation	NF-κB and MAPK pathway regulation	Anti-inflammatory functional ingredients
Redox-cycling compounds	α-Lipoic acid	Various nut matrices (trace levels)	Redox regeneration (LA/DHLA system)	Regeneration of other antioxidants (GSH, VC, VE)	Metabolic regulation; systemic antioxidant support
Metabolic regulators	Betaine	Walnut green husk	Indirect antioxidant via methyl donation and GSH support	Osmoprotection; redox balance maintenance	Nutraceuticals; metabolic health

### 3.1 Polyphenolic compounds

#### 3.1.1 Gallic acid

Gallic acid is rich in ortho-phenol hydroxyl groups, which can provide hydrogen ions to bind with surrounding free radicals, reducing the accumulation of free radicals and inhibiting oxidative reactions (49).

#### 3.1.2 Proanthocyanidins

**Figure 3:**
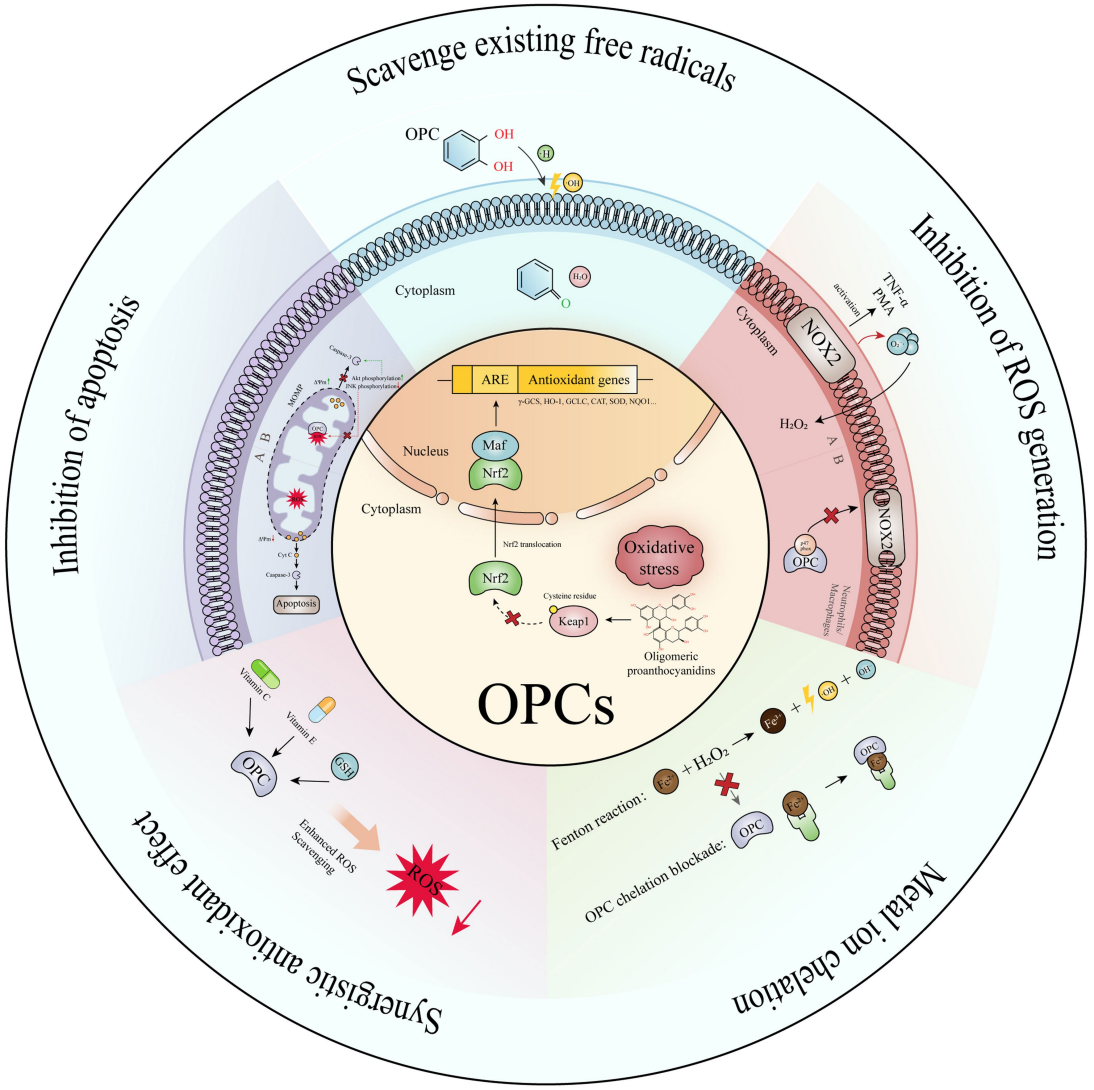
The antioxidant mechanism of proanthocyanidins.

Proanthocyanidins are a type of flavonoid polyphenolic compound found abundantly in various plants, renowned for their antioxidant, anti-inflammatory, and lipid metabolism-regulating properties. The antioxidant efficacy of proanthocyanidins is closely tied to their degree of polymerization, below the pentamer level are categorized as oligomeric proanthocyanidins, while those above are termed hyperpolymerized proanthocyanidins, which are not absorbed effectively by the body. Notably, walnut green peel is rich in oligomeric proanthocyanidins, and its antioxidant mechanisms are illustrated in Figure 3: (1) Scavenging Existing Free Radicals: The phenolic hydroxyl structure of oligomeric proanthocyanidins, particularly the neighboring hydroxyl groups, can directly donate hydrogen ions to neutralize free radicals. (2) Inhibiting Free Radical Production: These compounds can reduce the generation of reactive oxygen species by neutrophils and macrophages via the NADPH oxidase system. (3) Chelating Metal Ions: They chelate with metal ions such as Fe^2+^, Fe^3+^, and Cu^2+^, thereby preventing the conversion of superoxide (O₂^−^) and hydrogen peroxide (H₂O₂) into more reactive hydroxyl radicals (·OH). (4) Reducing Reactive Oxygen Species Levels: This is achieved through pathways involving protein kinase B and c-Jun amino-terminal kinase, which help to mitigate H₂O₂-induced apoptosis. (5) Synergistic Potentiation Among Antioxidants: Oligomeric proanthocyanidins work synergistically with other antioxidants. (6) Enhancing the Keap1-Nrf2-Antioxidant Response Element Pathway: Oligomeric proanthocyanidins can induce covalent modifications of Keap1 cysteine residues, leading to conformational changes that disrupt Keap1 dimers. This process increases the expression of antioxidant genes, thereby bolstering the endogenous antioxidant capacity. Under normal conditions, Nrf2 binds to Keap1 in the cytoplasm and is degraded by the cullin3 (Cul3) complex. However, during oxidative stress, Nrf2 dissociates from Keap1, translocates to the nucleus, and binds to V-Maf musculoaponeurotic fibrosarcoma (Maf). Nrf2 then recognizes and attaches to the antioxidant response element (ARE), activating the transcription of downstream antioxidant enzymes and target proteins, including *γ*-glutamylcysteine synthetase (γ-GCS), heme oxygenase-1 (HO-1), glutamate cysteine ligase catalytic subunit (GCLC), catalase (CAT), SOD, and NAD(P)H:quinone oxidoreductase (NQO1) (50, 51). (7) Regulating Multiple Signaling Pathways: By modulating pathways such as NF-κB, NADPH oxidase/ROS, AMPK/SIRT1, and MAPK, oligomeric proanthocyanidins activate corresponding signaling molecules, regulate gene expression levels, and influence the expression of various proteins, ultimately alleviating oxidative stress and exerting antioxidant effects. This comprehensive interplay contributes significantly to the potent antioxidant properties associated with oligomeric proanthocyanidins (52).

#### 3.1.3 Chlorogenic acid

Chlorogenic acid (53) is a condensed phenolic acid formed by the reaction between the carboxyl group of caffeic acid and the hydroxyl group of quinic acid. It is highly abundant in various plants and is known for its strong antioxidant properties. The mechanisms through which chlorogenic acid exerts its antioxidant effects include: (1) Chelating metal ions. (2) Directly scavenging free radicals. (3) Activating the Keap1-Nrf2-ARE pathway. (4) Inhibiting pro-oxidant enzymes and inflammatory pathways. (5) Protecting mitochondrial function. Regulating metabolism-related antioxidants. These mechanisms contribute to its potential health benefits.

#### 3.1.4 Naringin

Naringin (54) is a natural flavonoid glycoside recognized for its antioxidant and anti-inflammatory properties. Its antioxidant effects primarily involve the modulation of various antioxidant enzymes. Research has demonstrated that naringin can counteract the decrease in key antioxidant enzymes, including SOD, glutathione peroxidase (GPx), catalase, glutathione (GSH), and glutathione S-transferase (GST).

#### 3.1.5 Ellagic acid

Ellagic acid (55) is a naturally occurring polyphenolic compound with significant antioxidant activity and a complex variety of antioxidant mechanisms: (1) direct scavenging of free radicals. Ellagic acid’s own phenolic hydroxyl group can provide hydrogen atom radicals to generate stable ellagic acid radicals, thus terminating the free radical chain reaction, and it can also be oxidized itself to stable free radical intermediates by reducing the radicals to non-radicals or less reactive forms through electron transfer. In some polar environments, ellagic acid first loses protons to form phenoxylate anions, which neutralize free radicals by hydrogen atom transfer; (2) chelate metal ions. Ellagic acid chelates excessive metal ions and inhibits the Fenton reaction and lipid peroxidation, thereby reducing hydroxyl radical generation and oxidative damage to low-density lipoprotein; (3) inhibits oxidase activity. Ellagic acid inhibits the activities of cytochrome P450, xanthine oxidase, cyclooxygenase and NADPH oxidase, reduces O₂-·, H2O2 and blocks ROS generation at source. (4) regulates the intracellular antioxidant defense system. Ellagic acid promotes the expression of GSH, SOD and CAT by activating the Keap1-Nrf2-ARE pathway. Repairs oxidatively damaged biomolecules by promoting the activity of DNA repair enzymes (e.g., glycosylases) and protein hydrolases; (5) synergistic antioxidant effects. Ellagic acid has a synergistic effect with VC and VE, which can maintain the antioxidant capacity of VE and protect *β*-carotene from oxidative degradation by reducing VE free radicals.

#### 3.1.6 Ferulic acid

Ferulic acid (56) is a phenolic acid compound renowned for its potent antioxidant properties, which arise from its distinctive molecular structure and various mechanisms of action. (1) Molecular Structure Basis: The phenolic hydroxyl group of ferulic acid has the ability to donate hydrogen atoms, effectively neutralizing free radicals and forming stable phenoxy radicals. Additionally, the conjugated double bond and benzene ring stabilize radical intermediates, extending the antioxidant reaction’s chain termination capability through the *π*-electron off-domain effect. Furthermore, the methoxy group at the 3-position enhances the stability of phenoxy radicals via an electron donor effect. (2) Direct Mechanism of Action: Ferulic acid can neutralize free radicals through various processes, including hydrogen atom transfer, single electron transfer-proton transfer, and sequential proton loss electron transfer. It can also diminish ROS production by chelating metal ions. (3) Indirect Mechanism of Action: (a) Ferulic acid reduces intracellular ROS levels by inhibiting the activity of key enzymes such as NADPH oxidase, xanthine oxidase, and components of the mitochondrial respiratory chain. (b) It enhances the activity of antioxidant enzymes like SOD, GPx, and CAT by activating the Keap1-Nrf2-ARE signaling pathway. (c) Ferulic acid also works synergistically with other antioxidants, such as GSH and VC and VE, to create a robust antioxidant defense network. Notably, the combination of ferulic acid with VC and VE can enhance skin photoprotection by up to eightfold. Overall, ferulic acid serves as a multifaceted agent in the fight against oxidative stress, making it valuable in various health and skincare applications.

### 3.2 Flavonoids

Flavonoids are a class of polyphenolic compounds widely distributed in plants, exhibiting potent antioxidant and anti-inflammatory properties. They mitigate oxidative stress through direct scavenging of reactive oxygen species (ROS), chelation of transition metal ions, inhibition of pro-oxidant enzymes, and modulation of redox-sensitive signaling pathways such as Nrf2/Keap1, MAPK, and NF-κB. Collectively, these mechanisms protect biomolecules including lipids, proteins, and DNA, thereby preserving cellular integrity and function.

#### 3.2.1 Rutin

Rutin (57, 58), a flavonol glycoside, scavenges free radicals and inhibits lipid peroxidation, protecting cellular membranes and structures. Experimental evidence shows that rutin decreases malondialdehyde (MDA) levels while enhancing antioxidant enzyme activities such as superoxide dismutase (SOD) and catalase (CAT), reducing oxidative stress and improving neuronal function, for example in models of Alzheimer’s disease.

#### 3.2.2 Quercetin

**Figure 4:**
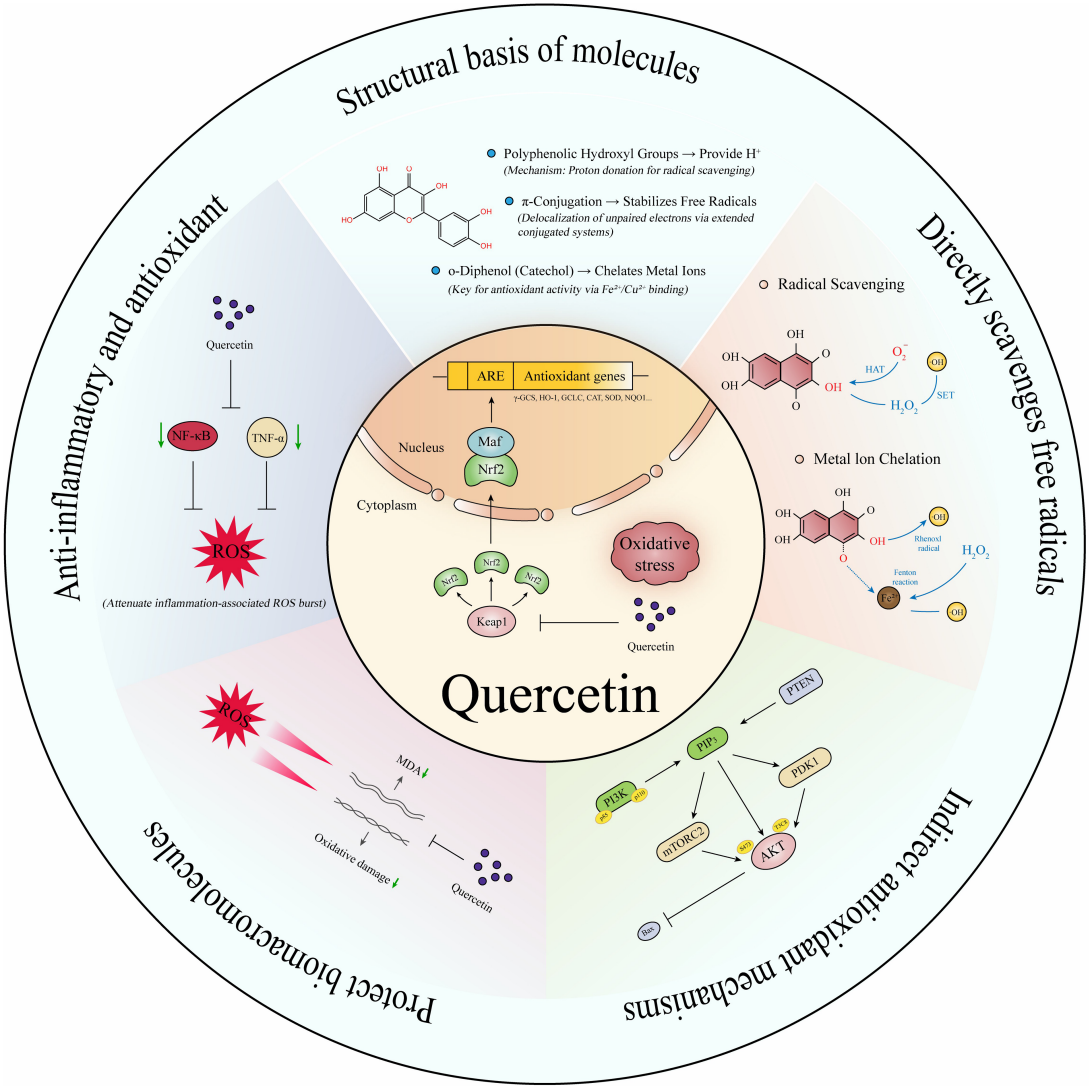
The antioxidant mechanism of quercetin.

Quercetin (59), a representative flavonol, exhibits multifaceted antioxidant mechanisms, as illustrated in Figure 4: (1) Molecular Structure: Its 3,3′,4′,5,7-pentahydroxyflavone structure provides polyphenolic hydroxyl groups for hydrogen donation, *π*-electron delocalization stabilizes free radicals, and the o-dihydroxyl group chelates metal ions, inhibiting the Fenton reaction. (2) Direct Antioxidant Mechanisms: (a) Free Radical Scavenging: Quercetin directly scavenges ROS through hydrogen atom transfer and single electron transfer. (b) Metal Ion Chelation: It chelates transition metal ions using its phenolic hydroxyl and carbonyl groups, which blocks the Fenton reaction and inhibits the generation of hydroxyl radicals. (3) Indirect Antioxidant Mechanisms: (a) Regulation of the Endogenous Antioxidant System: Quercetin enhances the expression of antioxidant enzymes such as GPx, Glutathione Reductase (GR), and *γ*-GCS by activating the Nrf2/ARE pathway, which promotes GSH synthesis and boosts cellular antioxidant capacity. It also enhances the activities of SOD, CAT, and peroxidase, facilitating the decomposition of ROS. (b) Regulation of Signaling Pathways: By inhibiting Keap1, quercetin activates Nrf2, promoting its nuclear translocation and upregulating antioxidant genes like HO-1 and NQO1. Furthermore, it activates the PI3K/AKT signaling pathway, inhibiting pro-apoptotic proteins such as Bax and supporting cell survival. Quercetin also reduces oxidative stress-induced cellular apoptosis by inhibiting the phosphorylation of p38 MAPK and JNK. (c) Inhibition of Pro-oxidant Enzyme Activities: Quercetin inhibits the activities of enzymes such as NADPH oxidase, xanthine oxidase, and cyclooxygenase, leading to a reduction in ROS generation. (4) Biomolecule Protection: Inhibits lipid peroxidation, lowers MDA, and protects DNA and proteins from oxidative damage. (5) Synergistic Anti-inflammatory Effects: By reducing ROS and inflammatory bursts, quercetin exerts integrated cytoprotective effects. Overall, quercetin is a multifaceted compound that not only provides strong antioxidant effects but also offers protective benefits to various biomolecules, contributing to overall cellular health.

#### 3.2.3 Other flavonoids

Other flavonoids, including Hyperin and Apigenin, share general antioxidant mechanisms with quercetin, such as ROS scavenging, Nrf2 activation, and enzyme regulation, but exhibit unique features (1) Hyperin (60) (quercetin-3-galactoside) stabilizes mitochondrial membrane potential, modulates nitric oxide synthases (cNOS, iNOS, tNOS), and enhances endogenous hydrogen sulfide production, contributing to anti-apoptotic and cardioprotective effects. (2) Apigenin (61) donates hydrogen atoms and electrons via its 4′-hydroxyl group, chelates metal ions, inhibits oxidase activity, and regulates multiple signaling pathways (Keap1-Nrf2-ARE, NF-κB, MAPK, PI3K/Akt), providing broad antioxidant and anti-inflammatory benefits.

Collectively, these flavonoids act synergistically to counteract oxidative stress and inflammation, with quercetin serving as a model compound illustrating multi-pathway regulation.

### 3.3 Other compounds

#### 3.3.1 Lipoic acid

Lipoic acid (LA), also known as *α*-lipoic acid, is a natural disulfide compound that plays a significant role in eliminating free radicals, thereby combating aging and various diseases. It is recognized for its excellent antioxidant properties and is classified as an endogenous antioxidant that can regenerate itself. In the body, lipoic acid can be converted into dihydrolipoic acid (DHLA). Both LA and DHLA possess strong antioxidant capabilities and work synergistically to enhance their effects. One of the unique features of lipoic acid is its ability to be absorbed by cells through the intestinal tract, exhibiting both fat-soluble and water-soluble properties. This dual solubility allows it to traverse the entire body effortlessly and reach various cellular components, functioning as a comprehensive, “all-purpose antioxidant.”

#### 3.3.2 Betaine

Betaine (62) is a naturally occurring quaternary ammonium compound detected in certain nut by-products, including walnut green husks, as summarized in Table 1. As a methyl donor in the methionine–homocysteine cycle, betaine contributes indirectly to antioxidant defense by supporting the synthesis of glutathione (GSH) and maintaining cellular redox balance.

Unlike classical phenolic antioxidants, betaine does not primarily act through direct radical scavenging. Instead, it enhances endogenous antioxidant capacity by modulating sulfur amino acid metabolism and preserving intracellular GSH levels. In addition, its zwitterionic structure confers osmoprotective and membrane-stabilizing effects, which may further protect cells from oxidative damage. Although its direct antioxidant potency is moderate, the presence of betaine in nut by-products may add functional value, particularly in the development of nutraceutical or functional food ingredients where metabolic regulation and cellular protection are relevant.

## 4 Integrated application of antioxidant components

**Figure 5:**
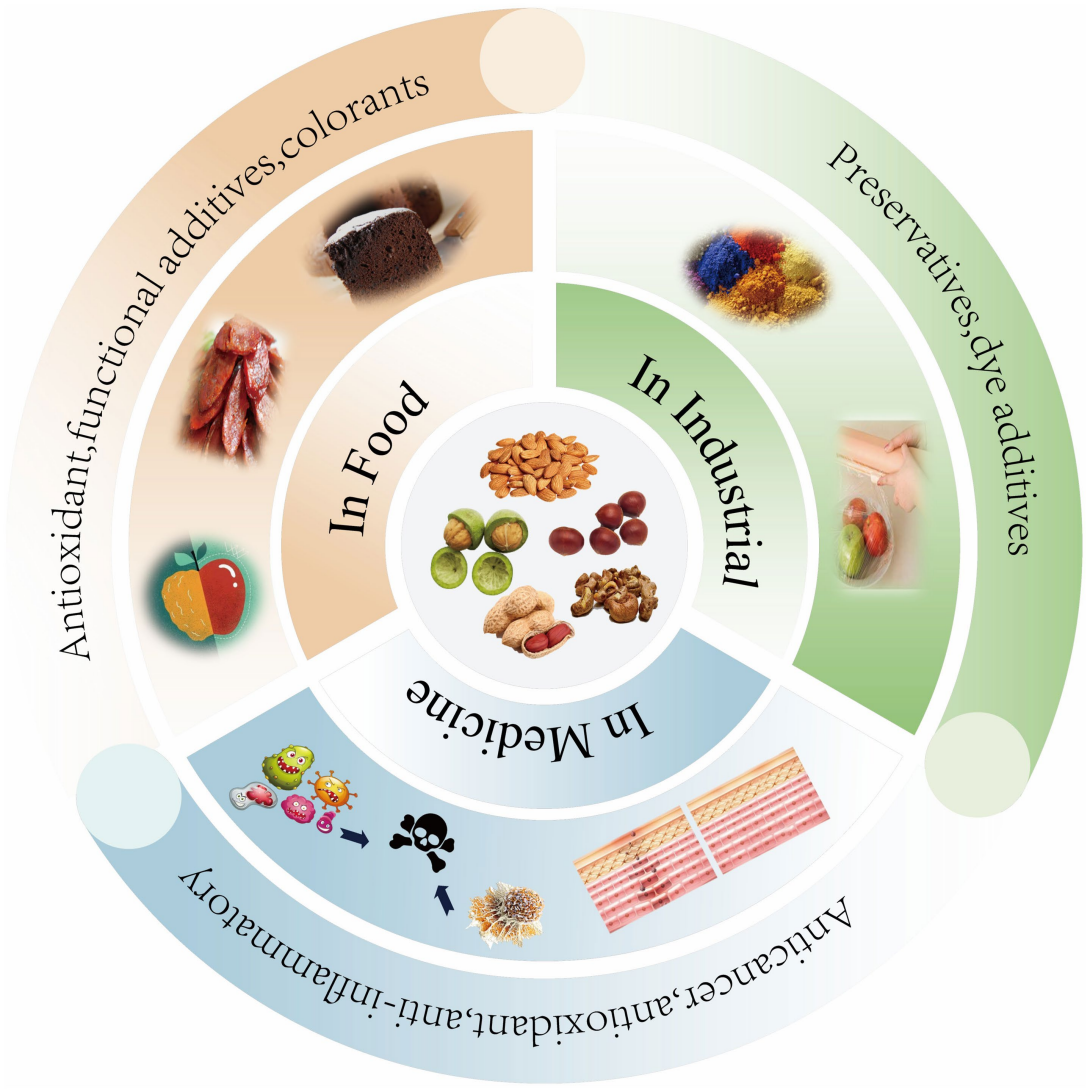
Comprehensive application of antioxidants.

The core antioxidant components identified in Section 2, including flavonoids, phenolic acids, tannins, and polysaccharides, constitute the principal bioactive matrix of nut processing by-products. As discussed in Section 3, these compounds exert their antioxidant effects through direct radical scavenging and redox-modulating mechanisms, forming the mechanistic basis for their functional performance. Building upon this compositional and mechanistic foundation, nut by-products demonstrate broad application potential in food preservation, functional ingredients, industrial materials, and biomedical contexts, as summarized in Table 4 and illustrated in Figure 5. This integration links molecular properties to value-added utilization within sustainable systems.

**Table 4:** Market applications and efficacy.

Field	Type	Raw materials	Function	Reference
In food	Colorants	Brown pigment extracted from chestnut shells	Easily soluble in water, strong coloring power, good stability, high temperature resistance, light resistance, acid and alkali resistance, can delay the aging of the cake, with antioxidant properties	(63)
		Colors extracted from green walnut peels	Strong water solubility, good light resistance, heat resistance, oxidation resistance, different colors at different pH values, used in color wine brewing and food coloring	(64)
	Functional additives	Cashew nutshell oil	Antioxidant, antimicrobial and anti-inflammatory properties as an alternative to chemicals in ruminant nutrition for the prevention of metabolic disorders	(65)
		Green walnut peel extract	Functional additive for meat processing to reduce weight loss, inhibit microbial growth, and improve organoleptic acceptance	(66)
	Antioxidants	Green walnut peel extract	Alternative to synthetic antioxidants with high antioxidant effect and low cost	(16)
		Peanut husk extract	Extracts added to lard can increase antioxidant capacity and have health effects such as lowering blood pressure and blood lipids	(33)
In industry	Preservatives	Green walnut peel extract	Preparation of active packaging films with enhanced mechanical properties, reduced permeability and improved antioxidant and antimicrobial activity	(69, 70)
		Cashew nut shell extract	Preparation of bioactive films with heat resistance, antioxidant activity and antimicrobial activity, non-toxic	(71)
		Peanut husk extract	Improvement of the film’s oxidation resistance, thermal stability and air permeability for air-conditioned packaging for fruit and vegetable storage	(72, 73)
		Almond peel extract	Extracts inhibit the growth of five food pathogens and antimicrobial properties confer antimicrobial activity on bioactive packaging	(74)
	Dye additives	Brown pigment extracted from chestnut shells	Good color fastness to many kinds of fabrics, low fastness to washing, high fastness to rubbing and light.	(75–82)
In medicine	Medicine	Chestnut shell extract	Polyphenols have anti-free radical, antimicrobial, and antitumor activities, which can be applied to the study of natural antitumor and antimicrobial drugs	(5) (88, 89)
		Green walnut peel extract	Polyphenols have antioxidant activity, help stop bleeding, and are candidates for thrombophilia treatment; polysaccharides are antioxidant, anti-inflammatory, and anti-cancer	(14) (83–86)

### 4.1 In food

#### 4.1.1 Colorants

The use of pigments is very common in the food industry, as foods with different colors can stimulate consumer appetite and perception. However, many pigments currently used on the market are chemically synthesized. Although these synthetic pigments are approved within regulatory limits, concerns regarding excessive intake, potential long-term health effects, and consumer perception have attracted increasing attention. China’s national standards impose restrictions on the use of certain synthetic additives in specific food categories, including baked goods. Therefore, the development and utilization of natural pigments are of increasing significance to the food industry, particularly in the context of clean-label and sustainability trends.

##### 4.1.1.1 Chestnut shell brown pigment

Chestnut shell pigment, which has a high content in chestnut shells, is a natural brown pigment characterized by high water solubility, strong coloring power, good stability, resistance to high temperature and light, and tolerance to acid–base conditions (maintaining stable hue at pH 4–14). Jin et al. (32) added chestnut shell browning pigment into cakes to explore its effect on cake quality and antioxidant activity. The results showed that chestnut shell brown pigment not only did not affect the quality and hue of the cake, but also delayed cake aging. Moreover, with the increase in pigment dosage, the scavenging rates of superoxide anions and DPPH free radicals were enhanced, indicating that it is a potential natural antioxidant.

##### 4.1.1.2 Colors extracted from walnut green peels

The natural pigments obtained from walnut green husks exhibits strong water solubility, light resistance, heat resistance, oxidation-resistance-reduction properties, and show different colors under different pH conditions: dark brown at pH 14.0–4.7, reddish-brown at pH 4.7–2.0, and yellow with reddish-brown precipitation at pH 2.0–1.0 (63). Ethanol has a color-enhancing effect on the pigment: adding a small amount of ethanol to a dilute solution of green husk pigment forms a deep wine red, which can be used in colored wine brewing. The brown pigment extract obtained by water extraction of air-dried walnut pericarp can be used for coloring foods such as soft candies, jellies, cakes and so on.

#### 4.1.2 Functional additives

##### 4.1.2.1 Cashew nutshell liquid

Functional oils are renowned for their substances with antioxidant, antimicrobial and anti-inflammatory properties and are used as alternatives to chemicals in ruminant nutrition. Cashew nutshell oil prevents metabolic disorders caused by high-concentration diets in ruminants to improve performance (63).

##### 4.1.2.2 Walnut green peel extract

In the meat processing industry, walnut green husk extracts can be used as functional additives serving as a low-cost and valuable source of phytochemicals. The effects of adding green husks extract on the partial characteristics of cooked sausages has been evaluated (64) and it was found that the green husks reduced weight loss during storage of cooked sausages. In addition, meat products with added walnut green husks showed less color deterioration during storage. The hardness of cooked sausages increased with the addition of walnut green husks, while elasticity and chewiness decreased. This significantly improved the sensory acceptance of odor and texture and inhibited microbial growth during storage.

#### 4.1.3 Antioxidants

Antioxidants play an important role in protecting oils and foods from oxidative deterioration. Many food antioxidants currently available on the market are synthetic compounds, such as BHA and BHT. Although these synthetic antioxidants are approved for use within established regulatory limits, their safety at high intake levels and long-term exposure has been subject to ongoing discussion. Some studies have reported potential adverse effects under excessive or non-compliant exposure conditions; however, when used within permitted limits, these additives are generally considered safe. Therefore, food scientists are increasingly exploring natural antioxidants as complementary or alternative options in response to consumer demand and sustainability considerations (65).

##### 4.1.3.1 Walnut green peel

Walnut green husks with high antioxidant activity and low price are considered as a source to replace synthetic antioxidants (16). Studies have been conducted on the antioxidant properties of walnut green husk extracts and their powders in sunflower oil. By comparing the antioxidant effect with the artificial antioxidant tert-butyl hydroquinone (TBHQ), it was found that the samples containing green husk extracts and its powder had stronger antioxidant properties, showing only weak oxidation under the same conditions. However, the use of walnut green husk extracts promoted the oxidation of edible oils. The experiments showed that the best antioxidant effect was achieved when the concentration of the green husk extract was 100 mg/kg, which effectively slowed down the oxidation process and was comparable to the effect of 200 mg/kg TBHQ.

##### 4.1.3.2 Peanut shells

Peanut shells as raw material for food antioxidant extraction has the advantages of low toxicity and higher consumer acceptability. Adding peanut shell extract to lard can not only improve the antioxidant capacity of lard, but also can play a role in lowering blood pressure, blood lipids and other health care effects (38).

##### 4.1.3.3 Chestnut shells

Huang et al. (66) used lard and peanut oil as substrates and employed an oven storage method to determine the POV values of the oils. They investigated the antioxidant activity of natural pigments from chestnut shells on the oils. The results indicated that the pigments from chestnut shells possess a certain antioxidant capacity against lipid oxidation. The best results were obtained when the pigment addition levels were 0.03 and 0.01% of the mass of lard and peanut oil, respectively.

Despite promising antioxidant performance in model oils and animal fats, most studies remain at the laboratory scale. Factors such as extract standardization, sensory impact, stability during high-temperature processing, and dose-dependent pro-oxidant effects require further investigation. In addition, regulatory approval and safety evaluation are essential before large-scale replacement of synthetic antioxidants can be realized.

### 4.2 In industry

#### 4.2.1 Preservatives

Bioactive food packaging is an innovative way to prevent food spoilage and microbial growth. Many researchers have focused on the application of plant extracts in active food packaging films, and the study of natural antioxidants made into active packaging films is imminent. When plant extracts are added to biodegradable films, the antioxidant, antibacterial, gas permeability, and structural properties of the films undergo certain changes.

##### 4.2.1.1 Walnut green peel extract

According to the study, an active packaging film can be prepared from chitosan/guar gum film matrix and walnut green husk extract. The results indicated that the mechanical properties of the composite film were significantly enhanced and the permeability of water vapor and oxygen was significantly reduced with the increase of the walnut green husk extract content (0–4%). When the extract dosage reached 4%, the DPPH radical scavenging activity of the composite film was significantly increased to 94.59%, which can effectively inhibit oxidation and microbial growth, showing great potential in food quality assurance and shelf-life extension (67); Xia et al. (68) extracted polyphenols from walnut green husks and incorporated them with curdlan and methylcellulose to make a novel edible composite film. The walnut green husk polyphenols significantly reduced the surface roughness of the composite film, improved its flexibility, and significantly improved the barrier capacity of ultraviolet light and water vapor, also significantly enhanced the antioxidant and antibacterial effects.

##### 4.2.1.2 Cashew nutshell extract

Bioactive films can be prepared by incorporating cashew nut shell liquid into sodium alginate matrix. It was found that the treatment with added cashew nut shell oil had better heat resistance and the permeability increased with increasing levels of cashew nutshell oil. The cashew nut shell oil films exhibit strong antioxidant activity and discrete antimicrobial activity. Ecotoxicity analyses showed that both the detected level of cashew nut shell oil and the produced films are non-toxic (69).

##### 4.2.1.3 Peanut shell extract

An experiment (70) explored the effects of peanut shell extracts on chitosan-based biodegradable films. The results showed that it improved the antioxidant capacity of the film, with high thermal stability, and its improved gas permeability and water vapor permeability, which are beneficial for developing active modified atmosphere packaging for postharvest fruit and vegetable storage.; Meng et al. (71) who addressed the effect of peanut shells and skins extracts on the antioxidant capacity, physical and structural properties of starch -chitosan active packaging film antioxidant capacity, physical structure properties. The results indicated that both peanut shell and peels have antioxidant activity, with peanut peels showing stronger antioxidant capacity.

##### 4.2.1.4 Almond shell extract

Sogut et al. (72) who studied the active extract of almond shells. They tested five foodborne pathogens, including *Escherichia coli*, *Pseudomonas aeruginosa*, *Listeria monocytogenes*, *Staphylococcus aureus*, and Salmonella spp. It was found that the extract inhibited the growth of all the five pathogens. The antioxidant property of the extracts can endow bioactive packaging with antibacterial activity, and it was confirmed that the antibacterial property could be maintained when the extracts were incorporated into sodium alginate films.

#### 4.2.2 Dye additives

##### 4.2.2.1 Chestnut shell coloring

The use of natural dyes for textile dyeing has become a new development trend, and chestnut shell pigment is a natural dye that can be applied on a large-scale industrial basis. At present, it can dye linen fabric, silk fabric, nylon fabric, antheraea pernyi silk fabric, cotton fabric, woolen fabric, wool fabric, cashmere fabric, with good dyeing effects.

Some researchers (73) found that chestnut shell pigments showed good dyeability on cellulosic fibers and the dyed fabrics had lower fastness to washing, but high rubbing and light fastness; Zhao et al. (74) dyed cellulose enzyme-pretreated linen fabrics with chestnut shell brown pigment, determined the optimal process, and the dyed linen fabrics had good color fastness to washing, rubbing, and sunlight; Zeng et al. (75) tested the coloration ability of chestnut shell pigment on silk fabrics and found that when dyeing at a constant temperature of pH 3–3.6 for 1 h without adding neutral electrolytes, silk fabrics with UV resistance could be developed. They also tested the dyeing performance of chestnut shell brown pigment on nylon fabrics, and the color fastness of the dyed fabrics was above grade 4, with good UV protection properties; Jia et al. (76) extracted pigments from chestnut shells and dyed them onto tussah silk fabrics, achieving good dyeing results and significant UV protection performance.; Wang et al. (77) used 5% chestnut shell pigment to dye chitosan-modified cotton fabrics, and the dyed cotton fabrics showed a yellowish-brown color, with good color fastness to soaping, perspiration, and light, as well as excellent antioxidant and UV resistance properties.; Dong et al. (78) determined the optimal dyeing process of chestnut shell brown pigment for wool fabrics, and the dyed wool fabrics obtained good dyeing effects, with color fastness to washing, rubbing, perspiration, and sunlight meeting the requirements; Ji et al. (79) used 10 g/L chestnut shell brown pigment to dye the wool fabrics using padding-steaming process. The pad-dyed wool fabrics showed light fastness grade 4, washing fastness above grade 4, dry rubbing fastness 4–5, and wet rubbing fastness 3-4; Zeng et al. (80) tested the dyeing performance of chestnut shell pigments on nylon fabrics. The results indicated that the optimal process for dyeing nylon with chestnut shell pigments is a pH of 3 to 3.6 and a temperature of 95 °C; the addition of neutral electrolytes reduces the dyeing percentage of the pigment; chestnut shell pigment exhibits good dyeing performance on nylon fabrics, with the colorfastness of dyed fabrics all exceeding grade 4. Mordanting has minimal impact on the colorfastness and color tone of chestnut shell pigment dyeing on nylon, and nylon fabrics dyed with chestnut shell pigment exhibit excellent UV protection properties.

Although nut by-product extracts show considerable potential in active packaging and textile dyeing, several practical challenges remain. These include variability in extract composition, potential migration of bioactive compounds into food matrices, long-term stability of antioxidant performance, and compliance with food contact material regulations. Comprehensive toxicological assessment and migration studies are necessary to ensure industrial feasibility.

### 4.3 In medicine

When the body is in a state of oxidative stress, excessive ROS are produced. Prolonged oxidative stress triggers an inflammatory response. Free radicals activate signaling pathways such as NF-κB and SP1, leading to the production of inflammatory factors (IL-1β, TNF-*α*), which ultimately lead to various chronic diseases. Therefore, nut by-products have attracted attention for their potential biomedical applications, particularly in relation to oxidative stress–associated conditions.

#### 4.3.1 Walnut green peel

Based on the antioxidant activity of polyphenolic compounds, walnut green husks polyphenols have been reported to exhibit hemostatic-related bioactivities in experimental models, suggesting their potential relevance in thrombotic disorder research (81); Walnut green husks polysaccharides have been shown in animal models to alleviate high-fat diet-induced abnormal body weight gain, metabolic disorders, liver injury, oxidative stress, chronic inflammation, vascular endothelial dysfunction, and modulation of gut microbiota (82). Existing *in vitro* studies have demonstrated cytotoxic effects of walnut green husks extracts on various cell types: the methanol extract of walnut green husks at a concentration of 9 mg/mL inhibited the proliferation of nasopharyngeal carcinoma CNE-2 cells (83); the ethanol extract at 7 mg/mL inhibited the proliferation of breast cancer MCF-7 cells (84); and the aqueous extract at 0.8–12.8 mg/mL inhibited the proliferation of HepG2 cells in a concentration-dependent manner (14). To evaluate the antioxidant capacity of walnut green husks aqueous extract, Li et al. (14) constructed a pathological model using t-BHP to induce oxidative stress and inflammation in HepG2 cells. The results study showed that treatment with walnut green husks aqueous extract led to the scavenging of ROS and the reduction of DNA damage. The aqueous extract inhibited the activation of the NF-κB pathway and reduced the production of the inflammatory cytokines TNF-*α* and IL-1β at 0.1 mg/mL.

#### 4.3.2 Chestnut shells

The biological activities of chestnut shell polyphenols in free radical scavenging, antibacterial activity, and antitumor effects indicate promising bioactivities, supporting further investigation into their potential as sources of natural antitumor and antibacterial agents.

Proanthocyanidins in chestnut shells have demonstrated antioxidant-related effects associated with cardiovascular protection in experimental studies by scavenging free radicals and inhibiting lipid peroxidation. After being absorbed into the bloodstream, proanthocyanidins enhance the total antioxidant capacity of serum and plasma, scavenge reactive oxygen species in plasma and arterial walls, thereby inhibiting the peroxidation of low-density lipoproteins, which may contribute to mechanisms relevant to atherosclerosis prevention (85–87). Proanthocyanidins exhibit cytotoxic effects on certain tumor cells. They demonstrate cytotoxic activity against human breast cancer cells (e.g., MCF-7, MDA-MB468), human lung cancer cells (e.g., A-427), and human gastric adenocarcinoma cells (e.g., CRL-1739). Proanthocyanidins have been reported to inhibit carcinogen-induced tumor lesions in experimental models (88–90). When comparing the antioxidant effects of VC, VE, VC + VB, and proanthocyanidins in protecting human oral keratinocytes from oxidative damage (oral cancer) induced by tobacco extract (STE), all antioxidants reduced STE-induced apoptosis, inhibited lipid peroxidation, and prevented DNA fragmentation. Among these, proanthocyanidins exhibited the strongest protective effect.

Adela et al. (91) conducted antimicrobial experiments on the extracts of inner and outer shells of chestnuts, and found that chestnut shell polyphenol extracts had a significant inhibitory effects on bacteria such as *Bacillus subtilis*, *Bacillus cereus*, and *Staphylococcus aureus*. Polyphenols are considered promising candidates for anticancer research due to their relatively low toxicity and selective cytotoxicity observed in preclinical studies. The combination of cytotoxic effects of polyphenols on cancer cells and protective effects on normal cells reflects their main advantages as anticancer drugs. Studies have shown (5) that chestnut shell extracts inhibit different human cancer cell lines, exhibiting highly cytotoxic effects on AGS cells (human gastric cancer cell system), HepG2 cells (human hepatocellular carcinoma cell line) and DU145 cells (tumor cell line).

While *in vitro* and animal studies suggest significant antioxidant and anti-inflammatory potential, most findings are preliminary. The bioavailability, metabolic transformation, and long-term safety of concentrated extracts remain insufficiently characterized. Furthermore, the high cytotoxicity observed at elevated concentrations highlights the need for dose optimization and careful safety evaluation before clinical translation.

## 5 Conclusion

Nut processing by-products, particularly walnut green husks, chestnut shells, and peanut residues, represent a significant yet underutilized reservoir of bioactive polyphenols and polysaccharides. This review highlights that while these residues exhibit potent antioxidant capacities in chemical systems, their nutritional and potential therapeutic relevance is governed by a complex structural–functional hierarchy. Specifically, low-molecular-weight phenolics offer immediate potential as natural food preservatives, whereas high-molecular-weight tannins and polysaccharides may contribute to broader systemic health benefits through the modulation of gut microbiota and cellular redox signaling.

However, moving from agricultural waste to standardized functional food ingredients requires addressing several critical gaps. First, the lack of standardized extraction protocols results in high variability in bioactive profiles, making cross-study comparisons and industrial scalability difficult. Future efforts must focus on developing “green” and cost-effective extraction technologies that can be transitioned from laboratory pilot scales to large-scale industrial production without compromising yield or bioactivity. Second, most current evidence relies on *in vitro* chemical assays, which fail to account for the bioavailability and extensive metabolic transformation of these compounds within the gastrointestinal tract. Systematic investigations into the digestion, absorption, and metabolic fate of these phytochemicals are essential to determine their actual physiological efficacy.

Furthermore, comprehensive safety assessments and toxicological evaluations remain a prerequisite for the inclusion of these by-products in the human diet, particularly for materials like cashew nut shell liquid which contains corrosive components. Future research should therefore prioritize mechanistic studies using cell-based and *in vivo* models to validate the safety and bioactivity of these antioxidants. Bridging these gaps—from industrial process optimization to clinical validation—is key to transforming nut by-products into validated, health-promoting bioresources that support a sustainable and circular food system.
